# Progression-Free Survival Prediction in Patients with Nasopharyngeal Carcinoma after Intensity-Modulated Radiotherapy: Machine Learning vs. Traditional Statistics

**DOI:** 10.3390/jpm11080787

**Published:** 2021-08-12

**Authors:** Ronald Wihal Oei, Yingchen Lyu, Lulu Ye, Fangfang Kong, Chengrun Du, Ruiping Zhai, Tingting Xu, Chunying Shen, Xiayun He, Lin Kong, Chaosu Hu, Hongmei Ying

**Affiliations:** 1 Department of Radiation Oncology, Fudan University Shanghai Cancer Center, Shanghai 200032, China; ronald.wihal92@hotmail.com (R.W.O.); 18211230042@fudan.edu.cn (Y.L.); 16211230041@fudan.edu.cn (L.Y.); k.fangfang@yahoo.com (F.K.); chengrun.du@yahoo.com (C.D.); 15211230047@fudan.edu.cn (R.Z.); xtingting2017@yeah.net (T.X.); 10301016003@fudan.edu.cn (C.S.); hexiayun1962@yeah.net (X.H.); drkonglin_shpic@sina.com (L.K.); drhucs@163.com (C.H.); 2 Department of Oncology, Shanghai Medical College, Fudan University, Shanghai 200032, China

**Keywords:** intensity-modulated radiotherapy, machine learning, nasopharyngeal carcinoma, survival prediction, traditional statistics

## Abstract

Background: The Cox proportional hazards (CPH) model is the most commonly used statistical method for nasopharyngeal carcinoma (NPC) prognostication. Recently, machine learning (ML) models are increasingly adopted for this purpose. However, only a few studies have compared the performances between CPH and ML models. This study aimed at comparing CPH with two state-of-the-art ML algorithms, namely, conditional survival forest (CSF) and DeepSurv for disease progression prediction in NPC. Methods: From January 2010 to March 2013, 412 eligible NPC patients were reviewed. The entire dataset was split into training cohort and testing cohort in a ratio of 90%:10%. Ten features from patient-related, disease-related, and treatment-related data were used to train the models for progression-free survival (PFS) prediction. The model performance was compared using the concordance index (c-index), Brier score, and log-rank test based on the risk stratification results. Results: DeepSurv (c-index = 0.68, Brier score = 0.13, log-rank test *p* = 0.02) achieved the best performance compared to CSF (c-index = 0.63, Brier score = 0.14, log-rank test *p* = 0.38) and CPH (c-index = 0.57, Brier score = 0.15, log-rank test *p* = 0.81). Conclusions: Both CSF and DeepSurv outperformed CPH in our relatively small dataset. ML-based survival prediction may guide physicians in choosing the most suitable treatment strategy for NPC patients.

## 1. Background

Nasopharyngeal carcinoma (NPC) is a type of head and neck cancer characterized by a distinctly unbalanced geographical distribution. Although accounting for only about 0.7% of all malignancies diagnosed in 2018, more than 70% of approximately 129,000 global new cases were found in East and Southeast Asia [1,2]. Since NPC is sensitive to ionizing radiation, radiotherapy is the primary treatment technique for non-metastatic condition. The widespread adoption of intensity-modulated radiotherapy (IMRT) and enhancement of chemotherapy strategies in the past decades have significantly improved the locoregional control of NPC, with decreased toxicities [3,4,5]. However, distant metastasis has emerged as the main cause of treatment failure of NPC, which accounts for about 70% of all NPC-specific mortality [6,7]. Furthermore, the existing TNM staging system, which often acts as the basis of treatment decision and prognostic outcomes, might not adequately represents tumor burden factors. There is a critical need of a multiparameter analysis to improve treatment decisions and predict patient outcomes, including clinical, pathological, and even biomolecular-related parameters.

Machine learning (ML) is a subset of artificial intelligence that uses various algorithms to automatically learn and adapt to new data without being explicitly programmed. Compared to conventional statistics, ML creates system that learns from data without using hypothesis-based assumptions. Additionally, ML has the ability to process high-dimensional data, which is perhaps beyond the capacity that biostatisticians can handle. Another advantage of ML over traditional statistics is that ML can learn non-linear interaction between variables. One main disadvantage of ML is the difficulty in interpreting the results generated by the models. However, there is a chance that this problem can be addressed in the future as more studies are working towards explainable ML [8].

Recently, ML has been applied for prognosis prediction in some types of cancer [9,10,11,12,13,14,15]. Notably, series of study applying ML for survival prediction of NPC have reported good performance [16,17,18,19,20]. Du et al. [18] built a ML model to predict 3-year disease progression based on 525 radiomics features extracted from magnetic resonance imaging (MRI) scan images and five clinical features. The study was conducted on 277 patients with non-metastatic NPC. The final model achieved an area under the receiver operatic characteristic (AUROC) curve of 0.80. Similarly, Zhang et al. [20] developed various ML models to predict local failure and distant failure in advanced NPC. A total of 970 radiomic features were extracted from MRI scan images of 110 patients. The study showed that the combined random forest model achieved the best performance with an AUROC curve of 0.8464 [20].

Most of the previous studies used accuracy, F-score, and AUROC as the evaluation metrics. These metrics are quantified in terms of disease status at a specific time point, where the time-to-event factor is not considered and, therefore, cannot adequately characterize survival outcomes. Cancer survival cannot be sufficiently described only with such binary outcomes and, thus, should incorporate a time-to-event element. The concordance index (c-index) and Brier score are two evaluation metrics that are considered suitable for prediction error for time-to-event data [21]. The C-index reflects model discrimination capacity to sort individuals from low to high value based on their risk, of which the values are between 0.5 (random prediction) and 1 (perfect prediction) [21]. The Brier score is a metric of both discrimination and calibration, of which the values range between 0 and 1, with a lower value representing better model accuracy [21].

Furthermore, there was no study that compares the performance of traditional statistics such as the well-known Cox proportional hazards (CPH) model with ML techniques in terms of the ability to predict survival for NPC patients. Recently, a study by Chen et al. [22] proposed a XGBoost model for risk stratification. However, the study did not make any comparison between their proposed model with traditional statistics-based models. Hence, there exists a vital need to investigate which model can achieve better performance for survival prediction at the individual level. In this study, we built two recently developed machine learning models, namely, conditional survival forest (CSF) and DeepSurv, based on the largest dataset of its kind while considering time-to-event outcome. The two models were compared with CPH using c-index, Brier score, and log-rank test.

## 2. Results

### 2.1. Patient Characteristics

**Figure 1:**
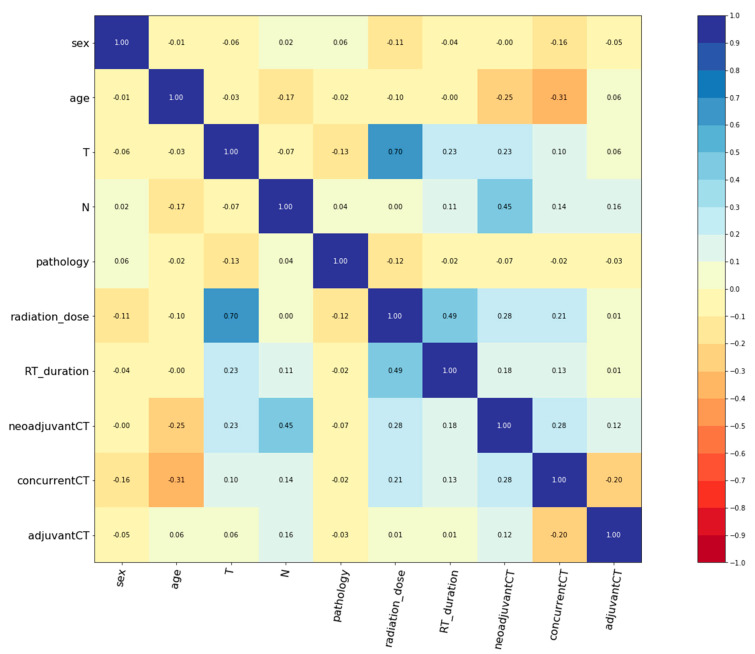
Correlogram illustrating the correlation between all variables. CT, chemotherapy; RT, radiotherapy.

Clinical characteristics of the 412 NPC patients are summarized in Table 1. The median age was 48 years old, ranging from 17 to 82 years old. There were 293 males (71.1%) and 119 females (28.9%). Based on the 7th edition of the American Joint Committee on Cancer staging manual, there were 8 (2.0%), 80 (19.4%), 199 (48.3%), and 125 (30.3%) patients with stage I, II, III, and IV, respectively. Among the study cohort, 56 patients (13.6%) received radiotherapy only, while the rest received combined chemoradiotherapy. Furthermore, Figure 1 revealed that there were little correlations between the chosen features.

### 2.2. Survival Analysis

**Figure 2:**
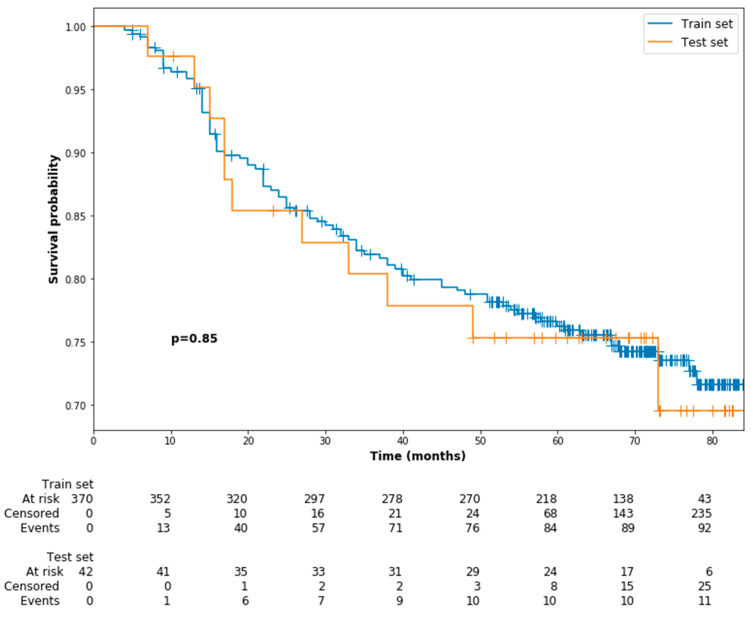
Kaplan–Meier survival curves of progression-free survival for the training cohort and testing cohort.

In general, the median follow-up period was 68 months, which ranged from 5 to 86 months. Throughout this period, 104 patients (25.2%) had disease progression, 61 patients (14.8%) were dead, and 18 patients (4.4%) were lost to follow-up. The 5-year PFS was 77.2%. During the modelling process, the dataset was entirely divided into training and testing cohorts, of 370 (89.8%) and 42 (10.2%) patients, respectively. No significant difference in PFS was observed between the training and testing cohorts (5-year PFS: 75.1% versus 73.8%, *p* = 0.85, Figure 2).

### 2.3. Univariate and Multivariate Analyses Based on CPH Model

Table 2 shows results from univariate and multivariate analyses based on CPH model. In Cox univariate analysis, advanced tumor and nodal classification were significantly associated with poor PFS. The hazard ratio (HR) of these variables on decreased PFS were 1.37 (95% confidence interval, 95% CI: 1.11–1.68, *p* < 0.005) and 1.53 (95% CI: 1.19–1.96, *p* <0.005), respectively. Moreover, radiation dose (*p* = 0.06) and neoadjuvant chemotherapy (*p* = 0.05) had trends toward significant correlation with PFS. In Cox multivariate analysis, tumor classification (HR: 1.59, 95% CI: 1.19–2.14, *p* <0.005), nodal classification (HR: 1.74, 95% CI: 1.34–2.26, *p* <0.005), and adjuvant chemotherapy (HR: 0.28, 95% CI: 0.08–0.94, *p* = 0.04) were found to be independently associated with PFS.

### 2.4. Performance Comparison

**Figure 3:**
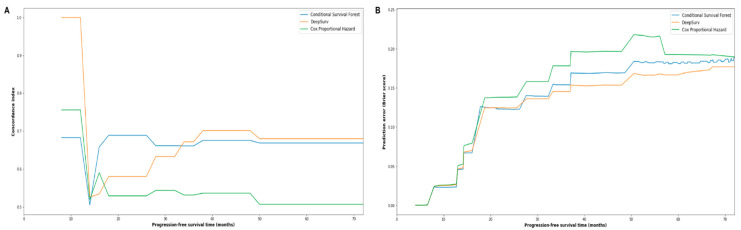
Plots of time-dependent concordance index (c-index) (**A**) and prediction error (Brier score) (**B**) comparing the three models.

The three models, namely, CPH, CSF, and DeepSurv, were trained on the training cohort and compared using the testing cohort. The optimized hyperparameters for CSF model were num_trees = 30, max_features = 1, and min_node_size = 2, while the optimized hyperparameters for DeepSurv were optimizer = ’adam’, lr = 0.0001, and dropout = 0.5, l2_reg = 0.0001. The prediction performance of different models was first compared in the testing cohort using c-index and Brier score. Figure 3A,B show the c-index and Brier score of the three models for PFS at different time points from 0 to 72 months. In terms of c-index, the CSF model achieved the highest performance in the first 30 months, while the DeepSurv model outperformed the CSF in the later timepoints. The integrated c-index of the CPH, CSF, and DeepSurv models was 0.57, 0.63, and 0.68, respectively. In terms of Brier score, the DeepSurv model overall delivered the highest performance at different time points. Furthermore, the integrated Brier score for CPH, CSF, and DeepSurv approaches was 0.15, 0.14, and 0.13, respectively.

**Figure 4:**
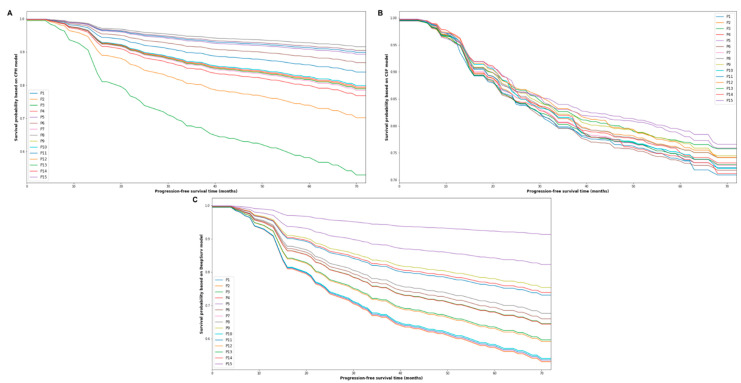
The progress-free survival predictions for 15 patients in the testing cohort based on the three models ((**A**) Cox proportional hazards model, (**B**) conditional survival forest, (**C**): DeepSurv).

**Figure 5:**
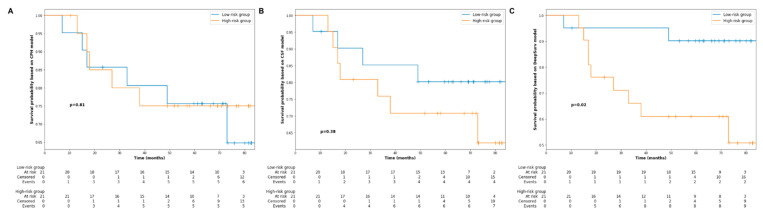
Kaplan–Meier curve estimates of progression-free survival for the testing cohort stratified into low- and high-risk groups depending on the median risk value output by the three models ((**A**) Cox proportional hazards model, (**B**) conditional survival forest, (**C**) DeepSurv).

Figure 4 shows the 72-month PFS probability of the first 15 individual patients in the testing cohort, based on the predictions made by the CPH (Figure 4A), CSF (Figure 4B) and DeepSurv (Figure 4C). Furthermore, the testing cohort was further stratified into a high-risk group and low-risk group based on the median risk predictions output by the three models. Figure 5 shows the Kaplan–Meier estimates of PFS for the testing cohort after the risk stratification. As can be seen, only the risk stratification based on the risk prediction made by the DeepSurv model achieved a significant difference in PFS between high-risk and low-risk groups (5-year PFS: 90.5% versus 61.9%, *p* = 0.02).

## 3. Discussion

Risk stratification and prognosis prediction play critical roles in clinical decision making, especially in the era of personalized medicine. Risk stratification helps to group patients into different categories based on their prognosis. Therefore, physicians can determine the most suitable treatment strategy based on the risk stratification result. There have been several studies that attempt to use conventional statistics and ML for these purposes. Among conventional statistics, the CPH model is the most extensively used statistical method in survival analysis. The main objective of this study was to compare the performance of CPH model with two state-of-the-art ML models, namely, CSF and DeepSurv. In general, our results showed that both CSF and DeepSurv models outperformed the CPH model in terms of c-index and Brier score. Furthermore, our study showed that the risk stratification based on the DeepSurv model was able to separate patients with different prognoses.

Currently, modelling of disease progression is often framed as a survival analysis task. The CPH model is the typical model of choice, since it involves time censoring and additional data as covariates. It is a semi-parametric regression method that describes the correlations between survival distribution and covariates [23]. Overall, CPH model estimates a function of time (log-risk function) as a linear combination of static covariates and baseline covariates. The main advantage of CPH, as a conventional statistical method, over ML models is its easy implementation and interpretation. However, CPH often relies on strong assumptions, such as proportional hazards assumption that each covariate has a constant multiplicative result in the hazard function [24,25]. These assumptions are not usually observed and are often overlooked in daily practice [24].

One ML approach that is often used to avoid the assumptions of CPH model is RSF, which is the adaptation of decision tree model. It is a fully non-parametric model, which works by constructing an ensemble estimate from base learners called trees for the cumulative hazard function [23]. After the model is built, classification is performed through assessing the instance with respect to base learners, and a decision is made by majority voting. One disadvantage of RSF is that there is a bias towards the inclusion of features with many split points [26]. CSF is the improved version of RSF that overcomes the disadvantage by means of conditional inference [26,27].

In recent years, several studies have been conducted to combine survival analysis with deep learning (neural networks) [28,29,30]. DeepSurv is a sophisticated deep learning approach proposed by Katzman et al. [28]. It is a deep feed-forward neural network with a configurable number of hidden layers, which estimates the effect of each individual’s covariates on their hazard rate with respect to parametrized weights of the network θ [28]. It has superior performance over CPH model, especially when dealing with non-linear data [28]. Furthermore, it can also handle the proportional hazards assumption of the CPH model.

There are some advantages of CSF and DeepSurv models over CPH model. First, both models are more flexible and free from prior assumptions [24]. Secondly, both models consider all available information on a specific area and are suitable for datasets with many features but only a few observations [31]. Another important advantage of both models is that they have the potential to analyze various types of data, such as medical images, laboratory test findings, and demographic data, which can be integrated into one platform for more ideal risk predictions [32].

One important advantage of this study is that we built prognostic prediction models using a time-to-event dataset. Right-censored data are quite common in cancer survival analysis, which means that follow-up ends before subjects experience a specific event, such as disease progression. As mentioned above, there are several studies that applied ML techniques for cancer prognosis prediction [16,17,18,19,20]. However, many ML approaches have an assumption that all patient outcomes are known (disease progression or no disease progression). Therefore, when applying ML methods for cancer survival analysis, one common strategy to overcome right-censored data is to split patient outcomes into categorical variables based on disease status at one specific time point. This approach does not consider the time-to-event factor and, therefore, may lead to bias [33]. In this study, we applied CSF and DeepSurv, two state-of-the-art approaches that allow survival prediction at the individual level and diminish the structural bias related to missing follow-up information.

The other advantage of this study is that the performances of the three models were investigated graphically with extensive metrics taking into consideration discrimination and calibration at different time points. Discrimination is a descriptor of the capacity of a predictive model to differentiate data at individual level, while calibration represents the agreement between the true outcomes and the predictions made by a predictive model at the population level. In this study, we used c-index as the discrimination metric and Brier score as the metric of calibration; both are suitable for time-to-event data analysis. Furthermore, we also compared the performances of the three models using the log-rank test by stratifying the testing set into high- and low-risk groups. Many similar studies in the past only adopted conventional metrics, such as accuracy and AUROC [16,17,18,19,20], which are not suitable for censored data. Moreover, to the best of our knowledge, this study is the first one to compare CPH, CSF, and DeepSurv using three different evaluation metrics in NPC.

Several limitations of this study should also be acknowledged. First, due to the retrospective nature of our study, the three models were built and evaluated based on a database from a single institution with limited observations, which hinders the generalizability of this study. However, a five-fold cross-validation approach was adopted to minimize bias and mimic external validation. Additionally, the present study acts as a guide to conduct a prospective multi-institutional trial hereafter. Secondly, this study only examined 10 features, while there might be various confounders that were not investigated. Thus, more comprehensive models that include more parameters, such as medical imaging, laboratory findings, and histopathological information, need to be evaluated in the future.

## 4. Materials and Methods

### 4.1. Study Cohort and Data

Institutional review board approval was obtained from Fudan University Shanghai Cancer Center prior to conducting this study. Written or verbal informed consent was not obtained from the participants due to the non-interventional retrospective nature of this study. Nevertheless, patient data were analyzed anonymously to maintain patient confidentiality.

Medical records of 412 newly diagnosed NPC patients treated with IMRT-based therapy at Fudan University Shanghai Cancer Center between January 2010 and March 2013 were retrospectively collected. The inclusion criteria were: (1) aged ≥ 16 years old; (2) Karnofsky performance status scale ≥ 80; (3) no evidence of distant metastasis and secondary primary cancer; (4) no prior malignancies or history of anticancer treatment. The exclusion criteria included: (1) history of neck dissection prior to chemoradiotherapy; (2) history of irradiation to head and neck; (3) incomplete chemoradiotherapy.

Relevant variables were collected, which can be classified into three different groups: patient-related, disease-related, and treatment-related variables. Patient-related data include age and gender; disease-related variables include World Health Organization (WHO) histological subtypes and tumor and nodal classification based on the 7th edition of the American Joint Committee on Cancer staging system; treatment-related features include radiation dose, radiotherapy duration, neoadjuvant chemotherapy, concurrent chemotherapy, and adjuvant chemotherapy status.

### 4.2. Treatment Protocol

Based on the standardized treatment protocol at our institution, radiotherapy was only recommended for patients with stage I, while combined radiotherapy was recommended for patients with stage II and above. Specifically, concurrent chemoradiotherapy was recommended for patients with stage II, while either concurrent chemoradiotherapy or neoadjuvant + adjuvant chemotherapy were recommended for patients with stage III and above.

All patients received radical IMRT. The radiation dose was 66–70.4 Gy to the planning target volume (PTV) of the gross tumor volume of nasopharynx (GTVnx), 66–70 Gy to the PTV of the gross tumor volume of positive neck lymph nodes (GTVnd), 60 Gy to the PTV of high-risk sites defined as clinical target volume 1 (CTV1), and 54 Gy to the PTV of low-risk sites defined as CTV2. The PTVs were delineated by adding 5 mm and 3–5 mm to the GTVs and CTVs, respectively. The radiation dose prescribed was given in 30–35 fractions.

The regimens given for neoadjuvant and adjuvant chemotherapy were mostly: (1) TPF regimen includes docetaxel, cisplatin, and 5-fluorouracil; (2) TP regimen includes docetaxel and cisplatin; (3) GP regimen includes gemcitabine and cisplatin. TPF and TP regimens were the first-line chemotherapy regimens, while GP regimen was chosen in case patients had the following conditions: peptic ulcer, upper gastrointestinal tract bleeding, cardiac diseases, diabetes, or food/drug allergy.

### 4.3. Follow-Up and Statistical Analysis

After completing the prescribed treatments, patients were followed-up every 3 months in the first 2 years and every 6 to 12 months thereafter. The outcome of this study was progression-free survival (PFS), of which definition is the duration between the time of initial chemotherapy or radiotherapy, whichever was earlier, and the date of locoregional recurrence, distant metastasis, or death from any cause.

All statistical analyses and drawings were performed using Python 3.7.4 (Scotts Valley, CA, USA). Correlation between variables was analyzed using the Pearson correlation coefficient. Actuarial rates for PFS and corresponding survival curves were generated using the Kaplan–Meier method and compared using the log-rank test. Univariate and multivariate analyses were performed using the Cox proportional hazards model. A two-sided *p* value less than 0.05 was considered statistically significant.

### 4.4. Modelling Process

**Figure 6:**
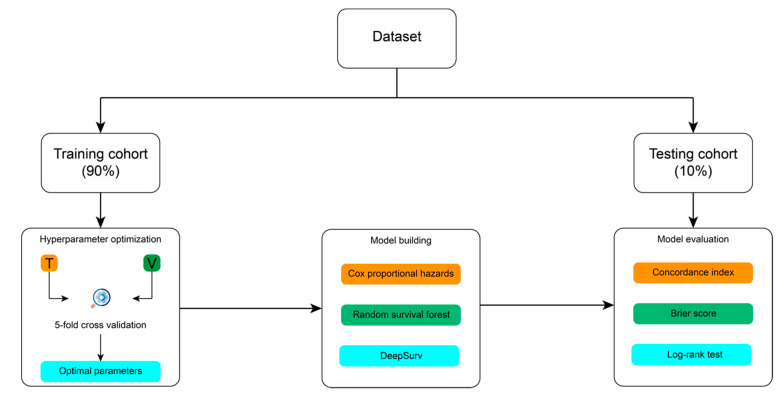
Illustration of the modelling framework.

Figure 6 summarizes the entire modelling process, from dataset splitting, hyperparameters optimization, model training, and model validation. Prior to developing CPH, CSF, and DeepSurv models, the dataset was divided into 2 separate cohorts. Nearly 90% of the whole study cohort was designated as the training cohort. We applied 5-fold cross-validation on the training cohort for model training and hyperparameter tuning. The remaining 10% of the entire study population was assigned as the testing set, which was utilized only once for final performance evaluation. During the splitting process, the dataset was stratified by disease progression status and, then, ranked by survival time to ensure both cohorts had roughly equal proportion of patients with disease progression and similar length of survival.

CSF was developed by Wright et al. [27] in 2017 for analyzing time-to-event data. It works by assembling the tree method and outputs the results by voting. CSF is considered as the improved version of random survival forest (RSF), since it corrects the bias in RSF model that results from favoring covariates with many possible split points [26]. There are three important hyperparameters in CSF, which are the number of trees (num_trees), number of variables to consider when deciding for best split (max_features), and minimum number of data points needed to be at leaf node (min_node_size). On the other hand, DeepSurv is a deep learning model developed by Katzman et al. [28] in 2016. It is a multi-layer feed forward neural network. The output is a negative partial log-likelihood parameterized by the weights of the network. In this study, we applied dropout and L2 regularization to prevent overfitting in our DeepSurv model and selected ReLU as the activation function. There are several important hyperparameters in DeepSurv, which are the optimizer (optimizer), learning rate (lr), dropout rate (dropout), and L2 regularization constant (l2_reg). All the hyperparameters mentioned above were optimized using the 5-fold cross-validation.

All the three models were developed using PySurvival python package [34]. Furthermore, model predictive performance was measured on the testing cohort using the c-index and Brier score. We also assessed the model performance using the log-rank test by stratifying the testing set into high- and low-risk groups depending on the risk value output by each model, where the median risk value was set as the threshold.

## 5. Conclusions

This study demonstrated the superior performances of machine-learning-based survival prediction models, namely, CSF and DeepSurv, compared to CPH as a conventional statistical method in terms of the c-index and Brier score. Moreover, our results showed that risk stratification based on the DeepSurv model was able to separate patients into high-risk and low-risk groups. The application of ML models for risk stratification may benefit patients through more personalized treatment strategies. Lastly, we recommend the use of comprehensive criteria, including discrimination, calibration, and interpretability, to assess ML approaches for NPC prognostication purpose.

## Figures and Tables

**Table 1:** Characteristics of all patients in the dataset (N = 412).

Characteristic	*n*	%
**Age (years)**		
Median	48	
Range	17–82	
**Sex**		
Male	293	71.1
Female	119	28.9
**WHO histological subtypes**		
Type 2 (non-keratinizing squamous cell carcinoma)	36	8.7
Type 3 (undifferentiated or poorly differentiated carcinoma)	376	91.3
**Tumor classification ^a^**		
T1	76	18.4
T2	135	32.8
T3	125	30.4
T4	76	18.4
**Nodal classification ^a^**		
N0	43	10.4
N1	147	35.7
N2	163	39.6
N3	59	14.3
**TNM stage ^a^**		
I	8	1.9
II	80	19.4
III	125	30.4
IV	199	48.3
**Radiation dose**		
≤66 gray	163	39.6
>66 gray	249	60.4
**Radiotherapy duration (days)**		
Median	44	
Range	33–61	
**Neoadjuvant chemotherapy**		
Yes	120	29.1
No	292	70.9
**Concurrent chemoradiotherapy**		
Yes	296	71.9
No	116	28.1
**Adjuvant chemotherapy**		
Yes	25	6.1
No	387	93.9

TNM, tumor–node–metastasis; WHO, World Health Organization. ^a^ Tumor–node–metastasis staging system based on the American Joint Committee on Cancer (7th edition).

**Table 2:** Cox proportional hazards regression for progression-free survival.

Variable	Univariate Analysis		Multivariate Analysis	
	HR (95% CI)	*p* Value	HR (95% CI)	*p* Value
Age	1.00 (0.99–1.02)	0.76	1.01 (1.00–1.03)	0.16
Sex	0.79 (0.49–1.27)	0.33	0.93 (0.59–1.47)	0.75
WHO histological subtypes	0.69 (0.37–1.30)	0.25	0.83 (0.45–1.54)	0.56
Tumor classification	1.37 (1.11–1.68)	<0.005	1.59 (1.19–2.14)	<0.005
Nodal classification	1.53 (1.19–1.96)	<0.005	1.74 (1.34–2.26)	<0.005
Radiation dose	1.00 (1.00–1.00)	0.06	1.00 (1.00–1.00)	0.81
Radiotherapy duration	1.00 (0.95–1.06)	0.92	0.96 (0.90–1.02)	0.16
Neoadjuvant chemotherapy	1.69 (1.01–2.83)	0.05	1.04 (0.60–1.79)	0.89
Concurrent chemoradiotherapy	1.48 (0.90–2.43)	0.12	1.00 (0.60–1.69)	0.99
Adjuvant chemotherapy	0.52 (0.16–1.65)	0.27	0.28 (0.08–0.94)	0.04

The regression was computed in the following ways: age as continuous variable; sex, male as reference; WHO histological subtypes, type 2 as reference; tumor classification, T1 as reference; nodal classification, N1 as reference; radiation dose as continuous variable; radiotherapy duration as continuous variable; neoadjuvant chemotherapy, no as reference; concurrent chemoradiotherapy, no as reference; adjuvant chemotherapy, no as reference. WHO, World Health Organization.

## Data Availability

The data presented in this study are available on request from the corresponding author. The data are not publicly available, as they contain information that are sensitive to the institution.
